# Supplementary material for: Unequal contribution of two paralogous CENH3 variants in cowpea centromere function
Source: Commun Biol. 2020 Dec 15;3:775. doi: 10.1038/s42003-020-01507-x (PMC7738545; doi:10.1038/s42003-020-01507-x)
Supplement: Supplementary file 1 — Supplementary Information [file 42003_2020_1507_MOESM1_ESM.pdf]

Supplementary Information

Tables

Supplementary Table 1: *Vigna* species used in this study.

Species	Section	Status	Origin	Genbank accession No.
Vigna unguiculata L. Walp. subsp. *unguiculata* cv. -gr. *unguiculata* (IT86D-1010)	Catiang	cultivated	Nigeria	IT86D-1010
Vigna unguiculata L. Walp. subsp. *unguiculata* cv. -gr. *unguiculata* (IT97K-499-35)	Catiang	cultivated	Nigeria	IT97K-499-35
Vigna unguiculata L. Walp. subsp. *unguiculata* cv. -gr. *biflora*	Catiang	cultivated	France	PHASEO NI147
Vigna unguiculata L. Walp. subsp. *unguiculata* cv. -gr. *unguiculata* (NI5)	Catiang	cultivated	USA	PHASEO NI5
Vigna unguiculata L. Walp. subsp. *unguiculata* cv. -gr. *unguiculata* (NI22)	Catiang	cultivated	Congo	PHASEO NI22
Vigna unguiculata L. Walp. subsp. *unguiculata* cv. -gr. *unguiculata* (NI139)	Catiang	cultivated	Cameroon	PHASEO NI139
Vigna unguiculata L. Walp. subsp. *unguiculata* cv. -gr. *unguiculata* (NI784)	Catiang	cultivated	India	PHASEO NI784
Vigna unguiculata L. Walp. subsp. *unguiculata* cv. -gr. *unguiculata* (NI1183)	Catiang	cultivated	China	PHASEO NI1183
Vigna unguiculata L. subsp. *alba*	Catiang	wild	Congo	PHASEO NI1656
Vigna unguiculata L. Walp. subsp. *unguiculata* var. *spontanea*	Catiang	wild	Kenya	PHASEO NI2210
Vigna unguiculata L. Walp. subsp. *baoulensis*	Catiang	wild	Ghana	PHASEO NI933
Vigna unguiculata L. Walp. subsp. *stenophylla*	Catiang	wild	Botswana	PHASEO NI1419
Vigna unguiculata L. Walp. subsp. *pawekiae*	Catiang	wild	Malawi	PHASEO NI1638
Vigna unguiculata L. Walp. subsp. *unguiculata* cv.-gr. *sesquipedalis*	Catiang	cultivated	Guyana	PHASEO NI126
Vigna aconitifolia (Jacq.) *Maréchal*	Aconitifoliae	cultivated	India	PHASEO NI41
Vigna angularis (Willd.) Ohwi & H.Ohashi var. *angularis*	Angulares	cultivated	China	PHASEO NI615
Vigna mungo Hepper var. *mungo*	Ceratotropis	cultivated	Australia	PHASEO NI515
Vigna radiata R.Wilczek var. *radiata*	Ceratotropis	cultivated	Iran	PHASEO NI4
Vigna reflexo-pilosa Hayata var. *glabra* (4x)	Angulares	cultivated	Philippines	PHASEO NI532
Vigna reflexo-pilosa Hayata var. *reflexo-pilosa* (4x)	Angulares	wild	Japan	PHASEO NI1684
Vigna trilobata Verdc.	Aconitifoliae	wild	Sri Lanka	PHASEO NI453
Vigna vexillata A.Rich.	Plectotropis	cultivated	Indonesia	PHASEO NI1859
Vigna umbellata (Thunb.) Ohwi & H.Ohashi var. *umbellata*	Angulares	cultivated	Congo	PHASEO NI204
Transgenic cowpea (IT86D-1010) AtDD45$_{pro}$:dsRED-Express	Catiang	Transgenic plant	Australia	Gursanscky et al. 2020

Supplementary Table 2: Primers and TaqMan based probes used in this study.

Name	5´-3´ Sequence
Vigna_CENH3F	ATGGCGAGAGTGAAGCACACGCCAGCTTCGC
Vigna_CENH3R	TCACCAAGGCCTTCCTATTCCTCCAAGCCTCCGAGCC
V.ungCENH3F	ACAGACTCAGGGAAGGAAGA
V.ungCENH3R	GGCAGCCGGGATAAGAAG
CENH3.1Probe	HEX-GGAACA+G+C+GGCG-IABkFQ
CENH3.2Probe	FAM-G+GAACA+A+T+GG+CG-IABkFQ
Ubiquitin28F	GATTCTGAAGGAGCTCAAGGAC
Ubiquitin28R	CCCATTATAGTTGCTTGCCAATG
Ubiquitin28Probe	ROX-AGAAAGACCCTCCAACCTCTTGCA-BBQ
GAPDH-F	CAATGATAGCTGCACCACCAACTG
GAPDH-R	CTAGCTGCCCTTCCACCTCTCCA
455 bp tandem repeats	Cy5-CAACGTGTGTTGATCCTTCT
pVuKB2F	CATGCATACACCAAGAAAGATC
pVuKB2R	TGTGCCTACTTTTGCTTGAGTC
721-bp tandem repeats	GGCTCACAACATTGTTTTCGC
721-bp tandem repeats	TTGGTATTCAGAGYCTGGST
1600-bp tandem repeats	CCGTCGATAAGAAGCAAATTCGT
1600-bp tandem repeats	ACTTGATATTTGTTACTTTCCAATGGT
PPT_F1	AGGAAAGGCCATCGTTGAAGAT
PPT_R1	TGTCTCGATGTAGTGGTTGACG
CAS9_F1	ACCTACCACGATCTCCTCAAGA
CAS9_R1	CTGAAACCTGAGCCTTCTGGAT
AtU6_26_F1	TGAACCGTAGCTTTCGTTTTCT
AtU6_26_R1	CAAGAAAGCTGGGTCTAG
Sg5CENH3.1F	AAGCCTGCGACAAGAAGTCG
Sg5CENH3.2F	TGTGATTGAAGCCTGTGACTAGAAGTAA
Sg5CENH3_common_R	CTGAAAATGACGAATCTCGCG
Sg3CENH3.1F	TTTCACTTCTCAAAACCAAACCCT
Sg3CENH3.1R	TCAACTCAAACGAAGAACGCG
Sg4CENH3.1F	TCACTTCTCAAAACCAAACCCTG
Sg4CENH3.1R	TCAGAACACCAAAAATGAATCAGCA
Sg4CENH3.2F	CACTCAAAGCCAGTCGCG
Sg4CENH3.2R	TCAGAACACCAAAAATGTATCAGCA
Drop off probe Sg3CENH3.1	HEX-TGAGCCCTTCTACGACTTCTTGTCG-BHQ1
Reference probe Sg3CENH3.1	FAM-ACGTACCCTGCGGCTCCTCTT-IABkFQ
Drop off probe Sg4CENH3.1	FAM-ACGTACCCTGCGGCTCCTCTT-IABkFQ
Reference probe Sg4CENH3.1	HEX-TCAACTCAAACGAAGAACGCGTTT-BHQ1
Drop off probe_Sg4CENH3.2	FAM-ACGTACCCTGCGGCTCCTCTT-IABkFQ
Reference probe_Sg4CENH3.2	HEX-TCAACTCAAACGAAGAAACGCAGTTT-BHQ1
Drop off probe Sg5CENH3_Common	HEX-TTCCTTCCCTGAGTCTGTGGCG-BHQ1
Reference probe_Sg5CENH3.1	FAM-AGCGCCGCTGTTCCTGG-IABkFQ
Drop off probe Sg5CENH3_Common	HEX-TTCCTTCCCTGAGTCTGTGGCG-BHQ1
Reference probe_ Sg5CENH3.2	FAM-AGCGCCATTGTTCCTGGCT-IABkFQ
nptII_F1	GCAGTTCATTCAGGGCACCG
nptII_R1	CGTGTCAAGTTGTGAGTGGC
pDEST_R4R3_F1	ACCATCGATTTACGCCAAGCTATCAACTTTG
pDEST_R4R3_R1	ACCGGTACCCGACGGCCAGTGAATTATCAAC
NOS_R1	CTCACCATACCGGTGATCTAGTAACATAGATGAC
At_pDD45_F2	TCTCGTAACTCAATCATCGC

Supplementary Table 3: Characterization of sequence CENH3.1 and CENH3.2 induced by CRISPR/Cas9 based genome editing.

Generation	Line name	CENH3.1	Allele	Sequence	CENH3.2	Allele	Sequence
T0	#5B1	Wild type	Chimera	GAAGAAGCAGCAGCAGCAGCGCCACAGA CTCAGGGAAGGAAGAA	Wild type	Chimera	AAGAAGAAGCAGCAGCGCCACAGACTCAGGGA AGGAAGAA
		-1		GAAGAAGCAGCAGCAGCAGCGCCACAGA C-CAGGGAAGGAAGAA	1		AAGAAGAAGCAGCAGCGCCACAGACCTCAGGG AAGGAAGAA
		-1		GAAGAAGCAGCAGCAGCAGCGCCACAGA- TCAGGGAAGGAAGAA	-4		AAGAAGAAGCAGCAGCGCCAC---- TCAGGGAAGGAAGAA
		1		GAAGAAGCAGCAGCAGCAGCGCCACAGA CCTCAGGGAAGGAAGAA	-7		AAGAAGAAGCAGCAGCGC------- TCAGGGAAGGAAGAA
		-4		GAAGAAGCAGCAGCAGCAGCGCCAC---- TCAGGGAAGGAAGAA	-6		AAGAAGAAGCAGCAGCGCCA------ CAGGGAAGGAAGAA
					-6		AAGAAGAAGCAGCAGCGCC------ TCAGGGAAGGAAGAA
					-9		AAGAAGAAGCAGCAGC--------- TCAGGGAAGGAAGAA
T1	#5B1-12	-1	Chimera	GAAGAAGCAGCAGCAGCAGCGCCACAGA- TCAGGGAAGGAAGAA	Wild type	Heterozygous	AAGAAGAAGCAGCAGCGCCACAGACTCAGGGA AGGAAGAA
		-4		GAAGAAGCAGCAGCAGCAGCGCCAC---- TCAGGGAAGGAAGAA	-6		AAGAAGAAGCAGCAGCGCCACAG------ GGAAGGAAGAA
		-48		GAAGAAGCAGCAGCAGCAGCG--------------- --------			
	#5B1-13	Wild type	Chimera	GAAGAAGCAGCAGCAGCAGCGCCACAGA CTCAGGGAAGGAAGAA	1	Biallelic heterozygous	AAGAAGAAGCAGCAGCGCCACAGACCTCAGGG AAGGAAGAA
		1		GAAGAAGCAGCAGCAGCAGCGCCACAGA CCTCAGGGAAGGAAGAA	-9		AAGAAGAAGCAGCAGC---------- TCAGGGAAGGAAGAA
		-1		GAAGAAGCAGCAGCAGCAGCGCCACAGA C-CAGGGAAGGAAGAA			
		-3		GAAGAAGCAGCAGCAGCAGCGCCAC--- CTCAGGGAAGGAAGAA			
		-9		GAAGAAGCAGCAGCAGCAGC--------- TCAGGGAAGGAAGAA			
		-34		GAAGAAACA---------------------------------A			
T2	Cenh3.1 KO	-1	Homozygous	GAAGAAGCAGCAGCAGCAGCGCCACAGA- TCAGGGAAGGAAGAA	Wild type	Heterozygous	AAGAAGAAGCAGCAGCGCCACAGACTCAGGGA AGGAAGAA
	#5B1-12.2				-6		AAGAAGAAGCAGCAGCGCCACAG------ GGAAGGAAGAA
	#5B1-12.3	Wild type	Wild type	GAAGAAGCAGCAGCAGCAGCGCCACAGA CTCAGGGAAGGAAGAA	Wild type	Chimera	AAGAAGAAGCAGCAGCGCCACAGACTCAGGGA AGGAAGAA
					-6		AAGAAGAAGCAGCAGCGCCACAG------ GGAAGGAAGAA
					1		AAGAAGAAGCAGCAGCGCCACAGACCTCAGGG AAGGAAGAA
					-4		AAGAAGAAGCAGCAGCGCCAC---- TCAGGGAAGGAAGAA
	#5B1-12.4	-1	Biallelic heterozygous	GAAGAAGCAGCAGCAGCAGCGCCACAGA- TCAGGGAAGGAAGAA	-6	Biallelic heterozygous	AAGAAGAAGCAGCAGCGCCACAG------ GGAAGGAAGAA
		A to T substitution		GAAGAAGCAGCAGCAGCAGCGCCACAGT CTCAGGGAAGGAAGAA	-2		AAGAAGAAGCAGCAGCGCCACAGAC-- AGGGAAGGAAGAA
T3	Cenh3.2 KO	A to T substitution	Heterozygous	GAAGAAGCAGCAGCAGCAGCGCCACAGT CTCAGGGAAGGAAGAA	-2	Homozygous	AAGAAGAAGCAGCAGCGCCACAGAC-- AGGGAAGGAAGAA

Supplementary Table 4: Characterization of *Cenh3.2* KO plants of cowpea induced by CRISPR/Cas9 based genome editing.

Genotype	Plant number	Number of pods per plant		Seed weight per plant (in g)	Length of selected high quality pods, n=5 (in cm)						Number of seeds per high quality pods, n=5												Seed weight of high quality pods (in g)
											Normal seeds						Shriveled seeds						
		High quality	Low quality	Seeds	1	2	3	4	5	average	1	2	3	4	5	average	1	2	3	4	5	average	Seeds
Cenh3.2 KO 4-3 (T4 generation)	1	17	0	20.4	17	14	14.5	12	14	14.3	10	9	10	7	9	9	2	0	0	1	4	1.4	8.1
	2	15	4	25.7	16.5	17.5	20	16	18	17.6	11	12	13	11	12	11.8	0	2	0	2	0	0.8	10.8
	3	16	2	23.1	14.5	16.5	17	16.5	18	16.5	8	14	12	13	11	11.6	1	0	2	0	2	1	10
	4	13	0	21	19	17	17.5	18.5	18	18	14	12	12	13	13	12.8	1	1	1	0	0	0.6	11.7
	5	12	2	19.1	16	18	20	15.5	17	17.3	10	12	15	12	9	11.6	4	3	0	2	4	2.6	10
	average	14.6	1.6	21.86	-	-	-	-	-	16.74	-	-	-	-	-	11.36	-	-	-	-	-	1.28	10.12
Cenh3.2 KO 4-14 (T4 generation)	1	16	0	20.6	16.5	15.5	16	16	17.5	16.3	11	10	12	9	12	10.8	1	1	0	5	2	1.8	9.2
	2	12	5	24.5	16	16	16	18	19	17	10	10	11	11	14	11.2	2	0	1	1	0	0.8	12.9
	3	18	1	18.3	18	18.5	18	16	15	17.1	11	11	12	11	9	10.8	0	4	0	3	1	1.6	7.5
	4	11	4	25.5	15	18	17	19	19	17.6	8	13	13	12	15	12.2	3	0	0	2	0	1	11.2
	5	15	1	19.4	16.5	18	17	16	14.5	16.4	15	12	11	8	9	11	0	0	0	4	1	1	10.8
	average	14.4	2.2	21.66	-	-	-	-	-	16.88	-	-	-	-	-	11.2	-	-	-	-	-	1.24	10.32
Wild type	1	17	5	22.1	18	16	15.5	16	16	16.3	14	10	11	10	11	11.2	0	0	2	3	1	1.2	10
	2	22	5	25	17	15.5	13.5	16	15	15.4	12	10	9	10	9	10	0	0	0	1	0	0.2	9.1
	3	16	6	24.2	15.5	17	18	16.5	17	16.8	10	11	14	11	13	11.8	1	2	0	0	0	0.6	11.4
	4	18	0	24.9	16.5	18.5	17.5	17	15	16.9	12	15	12	11	11	12.2	2	0	0	4	0	1.2	10.5
	average	18.25	4	24.05	-	-	-	-	-	16.35	-	-	-	-	-	11.3	-	-	-	-	-	0.8	10.25

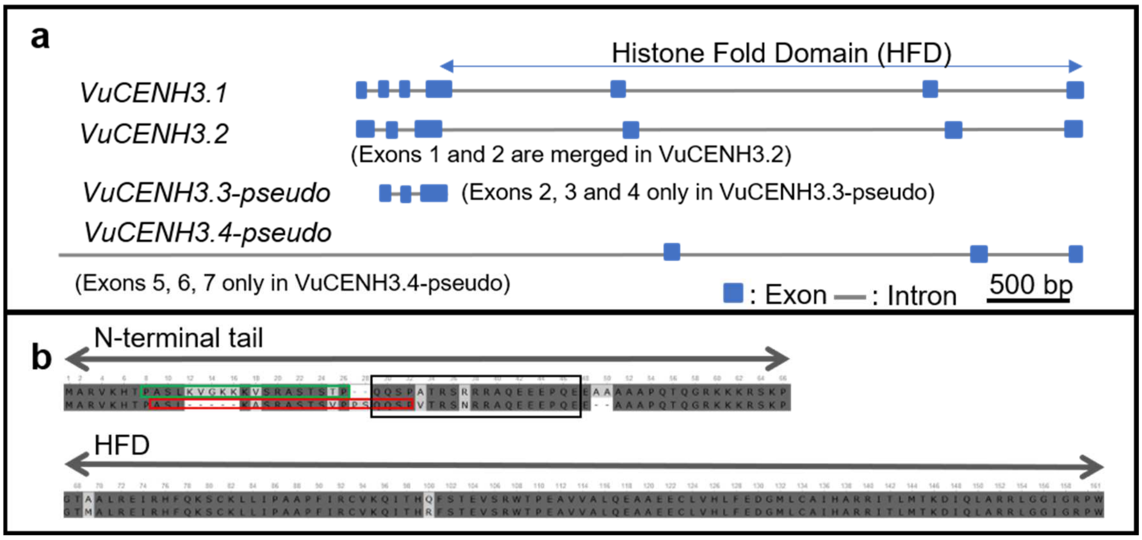

Supplementary Fig. 1: Schematic illustration of the cowpea CENH3 gene exon and intron structure.

a Alignment of CENH3.1 and CENH3.2 amino acid sequences. **b** Green, red and black boxes indicate the position of the peptides used for the generation of CENH3.1, CENH3.2 and CENH3 common antibody, respectively.

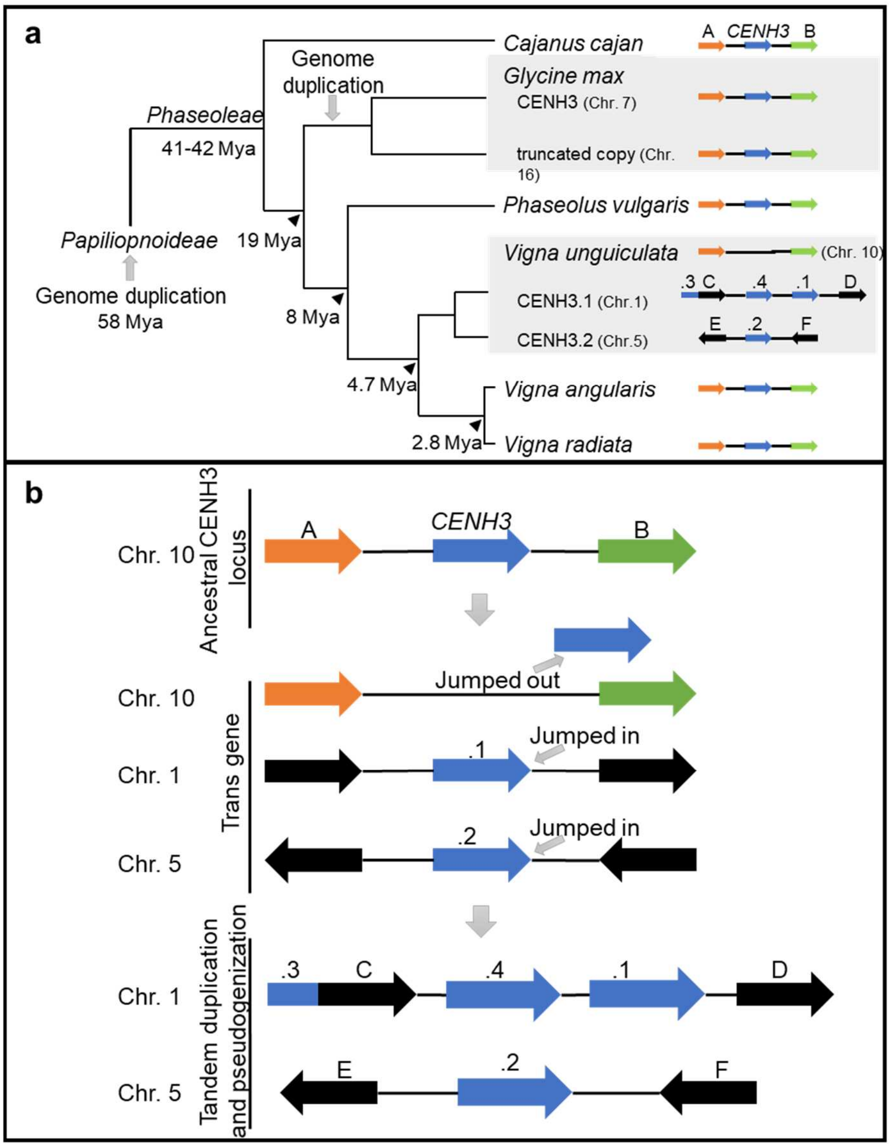

Supplementary Fig. 2: Phylogenetic tree based on the CENH3 amino acid sequence, with a scheme of the chromosomal *CENH3* gene locus on the right of each species.

a Genome duplication events and node ages are based on (Lavin et al. 2005; Cannon et al. 2010; Kang et al. 2014). **b** Schemata of possible mechanism of CENH3 duplication in cowpea genome. Blue arrows indicate *CENH3* gene locus and nearby genes indicated by different colours as A: Rhodanese-like domain-containing protein, B: WRKY transcriptional factor, C: unknown (exosome complex exonuclease), D: unknown, E: calcium-dependent lipid-binding domain-contained protein, F: 60S ribosomal protein L18A, respectively.

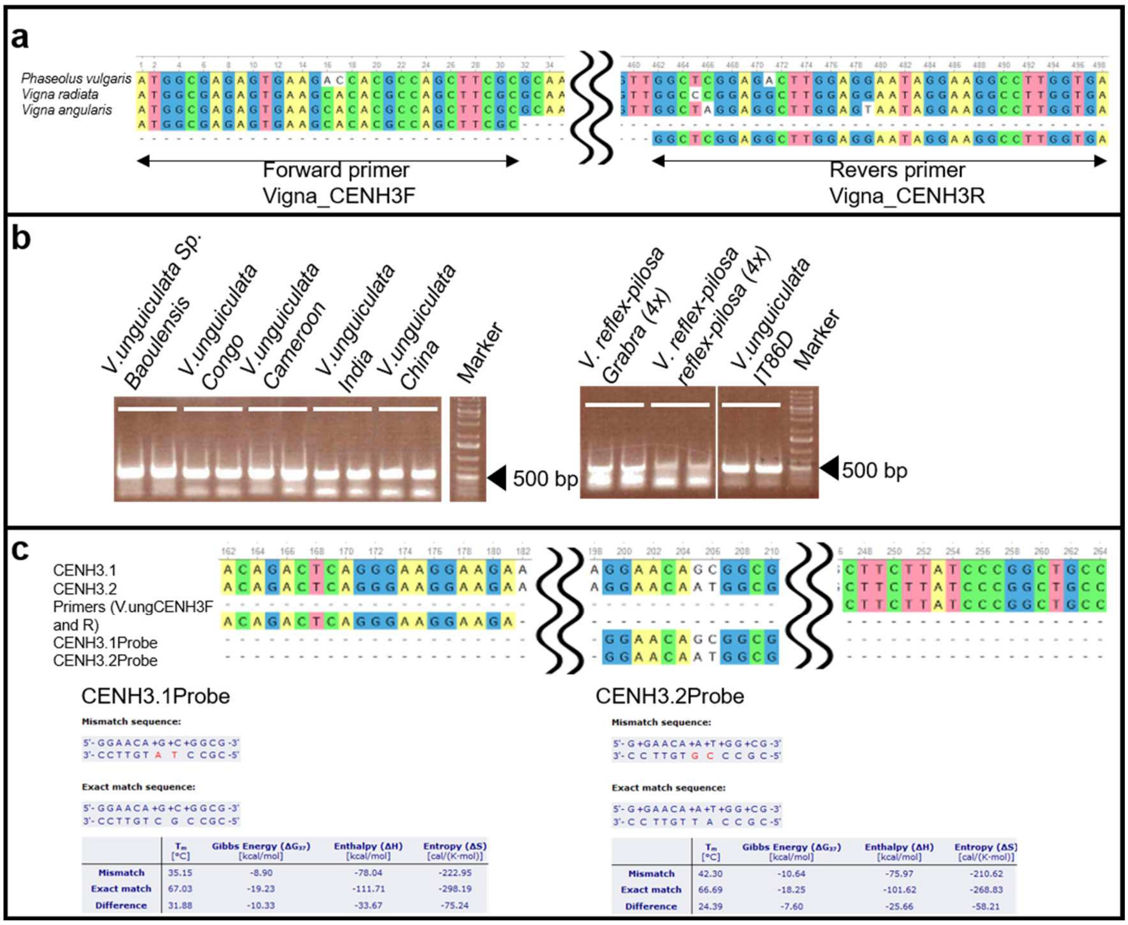

Supplementary Fig. 3: Primer design for *CENH3* genes.

a Multiple alignments of cDNA sequence from *Phaseolus vulgaris, Vigna radiata* and *Vigna angularis* for designing of RT-PCR primer sequences for cloning of *CENH3* sequences from *Vigna* species. **b** RT-PCR products from six *V. unguiculata* accessions and two V. reflex-pilosa accessions confirm the *CENH3* amplification. **c** Multiple alignments of cDNA sequences of cowpea *CENH3.1*, *CENH3.2,* primers for qRT-PCR (V.ungCENH3F and R), and TaqMan probes (CENH3.1Probe and CENH3.2Probe) and annealing temperature calculation of probes.

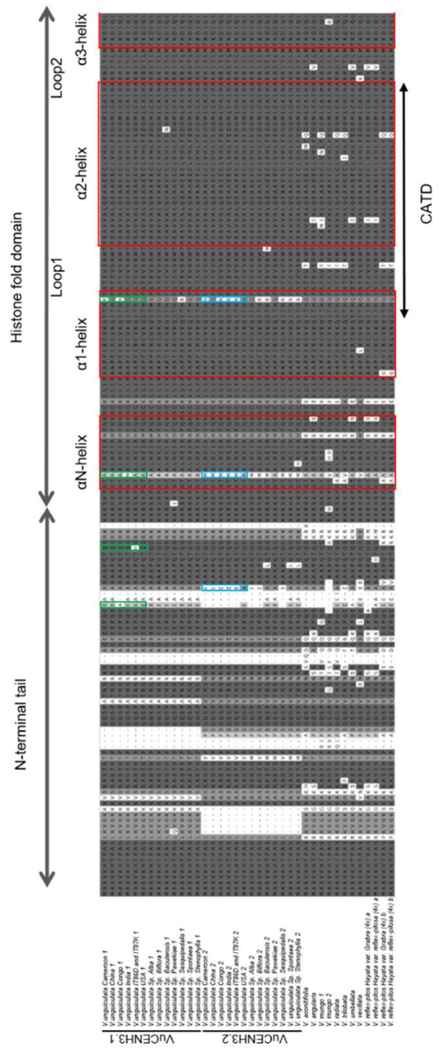

Supplementary Fig. 4: Diversity of CENH3 in *Vigna* species.

Multiple alignments of CENH3 proteins from different cowpea genotypes of different origin (*V. unguiculata* -Cameroon, -China, -Congo, -India, -IT86D-1010, -IT97K-499-35 and -USA), different subspecies or varietas of *V. unguiculata* (*alba, biflora, baoulensis, pawekiae, sesquipedalis, spontanea* and *stenophylla*), different diploid *Vigna* species (*V. aconitifolia, V. angularis, V. mungo, V. radiata, V.trilobata, V. umbellate* and *V. vexillata*), and different tetraploid *V. reflexo-pilosa genotypes* (*V. reflexo-pilosa var glabra* and *V. reflexo-pilosa var. reflexo-pilosa*). Conserved CENH3 domains are indicated with red-boxes. CENH3.1 and CENH3.2 amino acid mutations in cowpea accessions indicated with green- and blue-boxes, respectively.

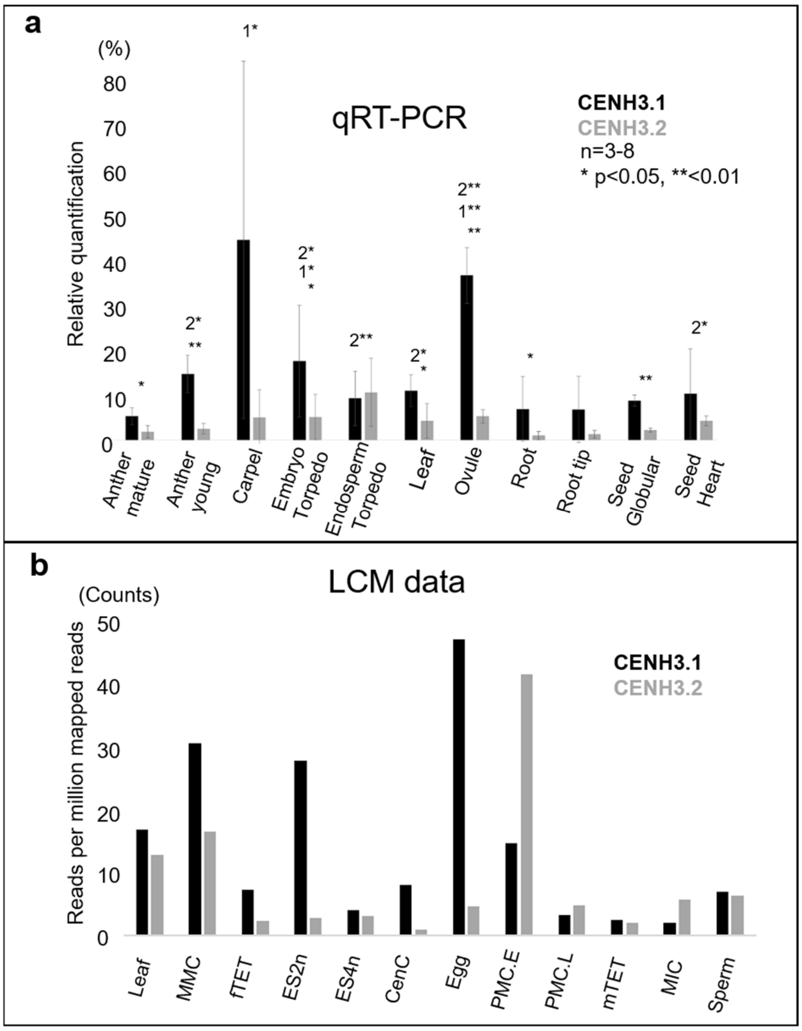

Supplementary Fig. 5: Gene expression patterns of *CENH3.1* and *CENH3.2* in different tissue and cell types of cowpea.

a qRT-PCR analysis using RNA isolated from different tissues of cowpea. Transcript levels of each gene were normalized to *Ubiquitin28* using the ΔΔCt-method (Schmittgen and Livak, 2008). Relative quantification values are calculated by fold change to root *CENH3.2*. **b** RNA-sequencing using RNA isolated from laser capture microdissected cell types of cowpea. Leaf, MMC-megaspore mother cell, fTET-female tetrads, ES2n-embryo sac (2 nuclei), ES4n-embryo sac (4 nuclei), CenC-central cell, egg, PMC.E-early pollen mother cell, PMC.L-late pollen mother cell, mTET-male tetrads, MIC-microspore, sperm. Significant difference between *CENH3.1* and *CENH3.2* within tissue types was indicated *: $p < 0.05$, **< 0.01 (t-test). Significant difference compares to root with different tissue types was indicated for *CENH3.1* (1*: $p < 0.05$, 1**< 0.01) or *CENH3.2* (2*: $p < 0.05$, 2**< 0.01). Indicate SD in figure (**a**).

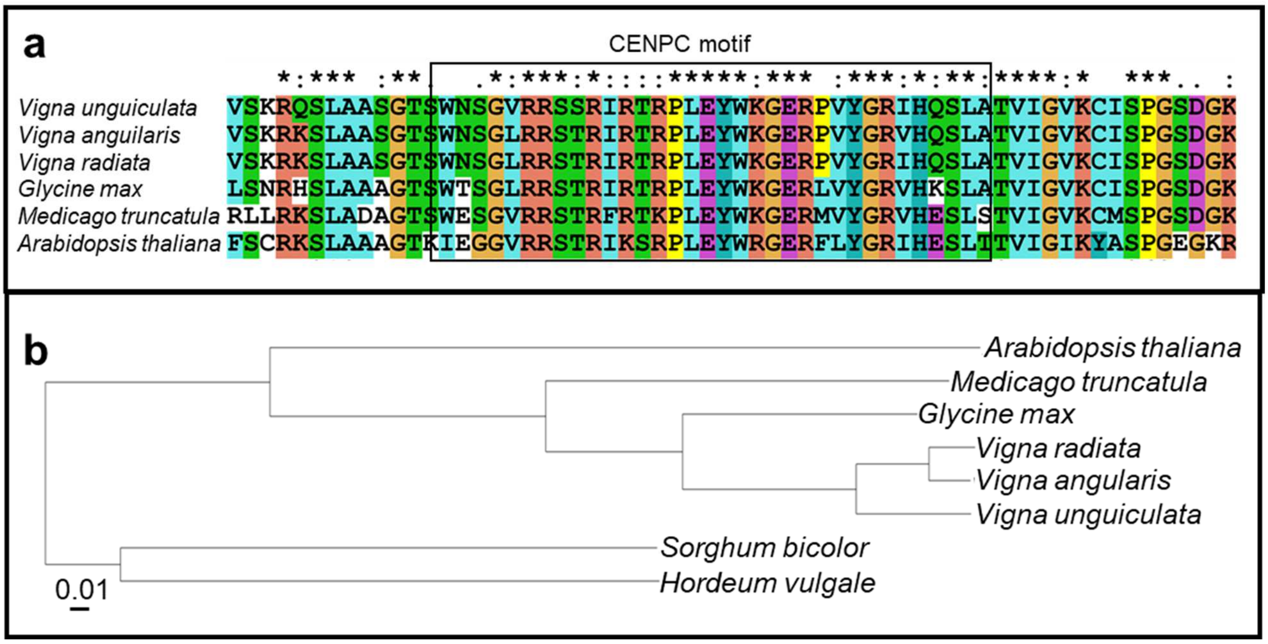

Supplementary Fig. 6: Identification of cowpea *CENPC*.

Alignment of partial CENPC proteins of *A. thaliana, G. max, M. truncatula, V. angularis, V. radiata* and *V. unguiculata* (a). Phylogenetic tree based on the full length CENPC proteins of *A. thaliana, G. max, H. vulgale, M. truncatula, S. bicolor, V. angularis, V. radiata* and *V. unguiculata*.

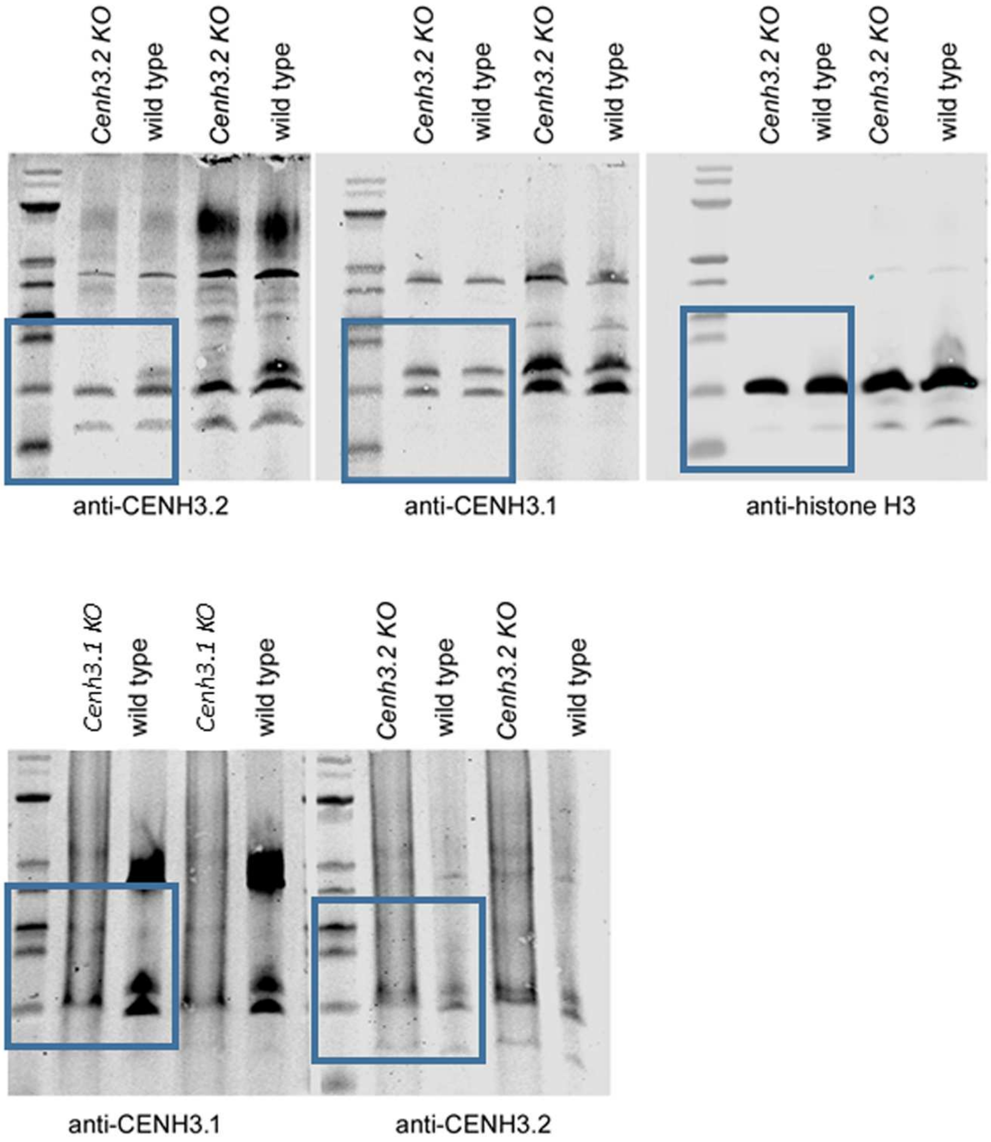

Supplementary Fig. 7: Uncropped comparative Western blots.
Western blots of isolated nuclear proteins from wild type, *Cenh3.1* and *Cenh3.2* KO plants with anti-CENH3.1, anti-CENH3.2 and anti-histone H3. Regions used for Figure 2c, d are indicated.

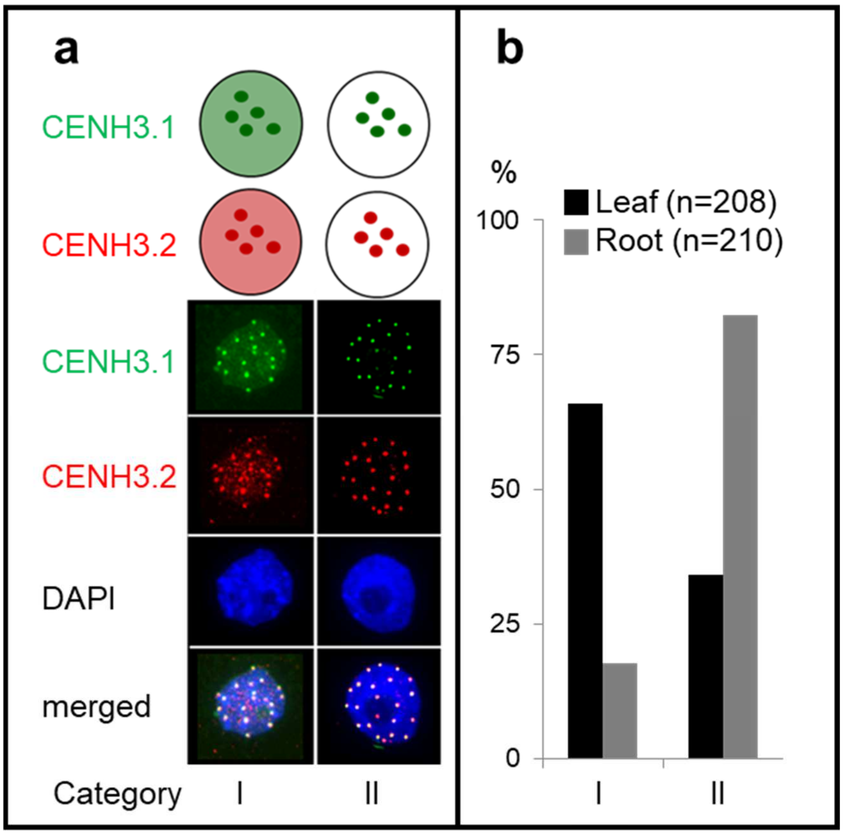

Supplementary Fig. 8: Distribution of CENH3.1 and CENH3.2 immunosignals in isolated nuclei from leaf and root tissues of cowpea.
a Two categories are determined based on the configurations of CENH3 signals. Category I: Centromeric CENH3.1 and CENH3.2 signals colocalize and nuclei reveal disperse immunosignals. Category II: Centromeric CENH3.1 and CENH3.2 signals colocalize without dispersing signals. Typical examples are shown in (a). **b** Quantification of categorized leaf (n=208) and root (n=210) nuclei. Black bar: leaf nuclei, grey bar: root nuclei.

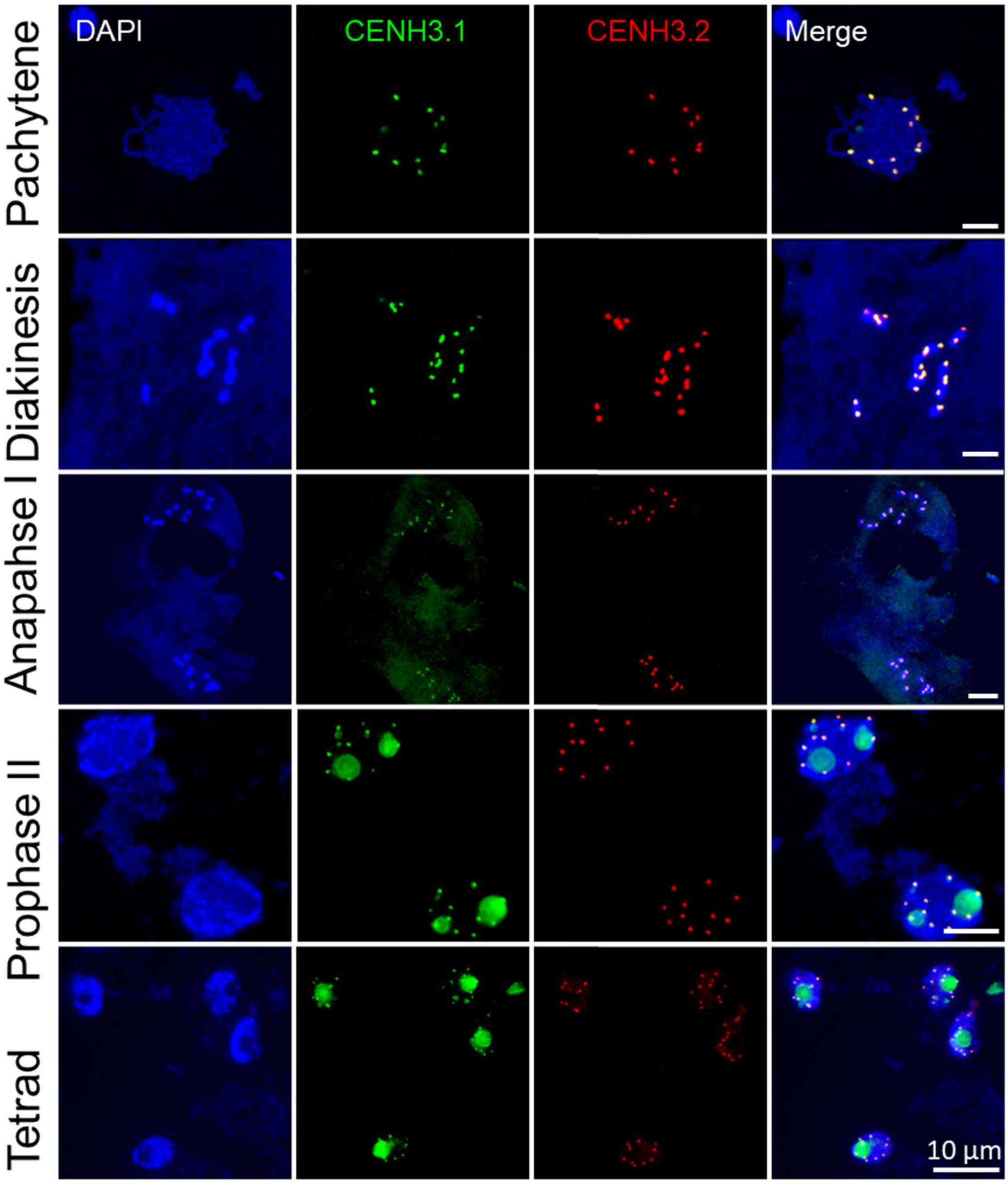

Supplementary Fig. 9: Centromeric CENH3.1 and CENH3.2 colocalize during all stages of male meiosis in cowpea.

Distribution of CENH3.1 (green) and CENH3.2 (red) are shown in pachytene, diakinesis, anaphase I, prophase II and tetrad cells.

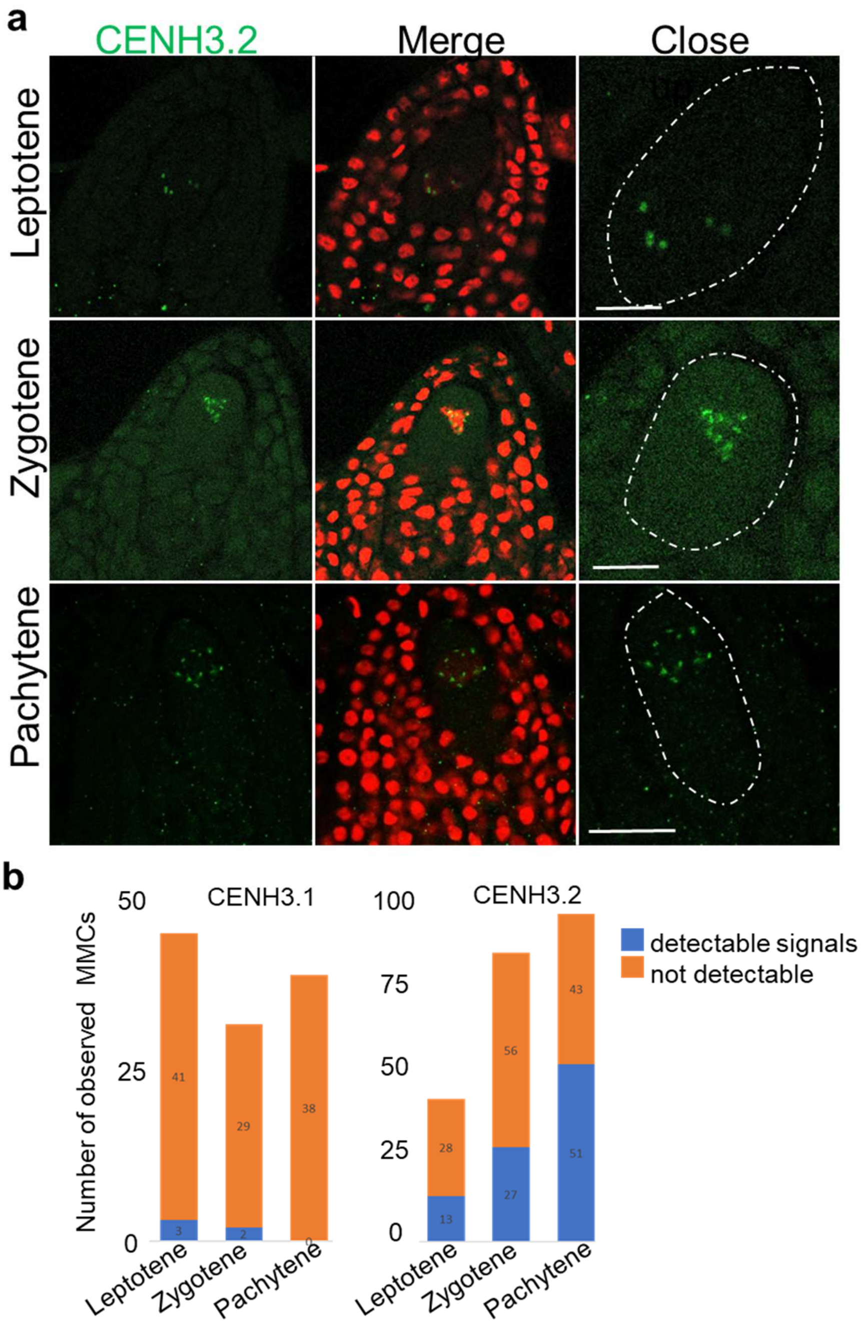

Supplementary Fig. 10: Distribution of CENH3.1 and CENH3.2 during female meiosis of cowpea tissue sections.

a CENH3.2 immunostaining signals (green) at leptotene, zygotene and pachytene. Nuclei were counterstained with propidium iodide (red). **b** Frequency of observed CENH3.1 and CENH3.2 signals in leptotene, zygotene and pachytene cells. Bars equal 10 µm.

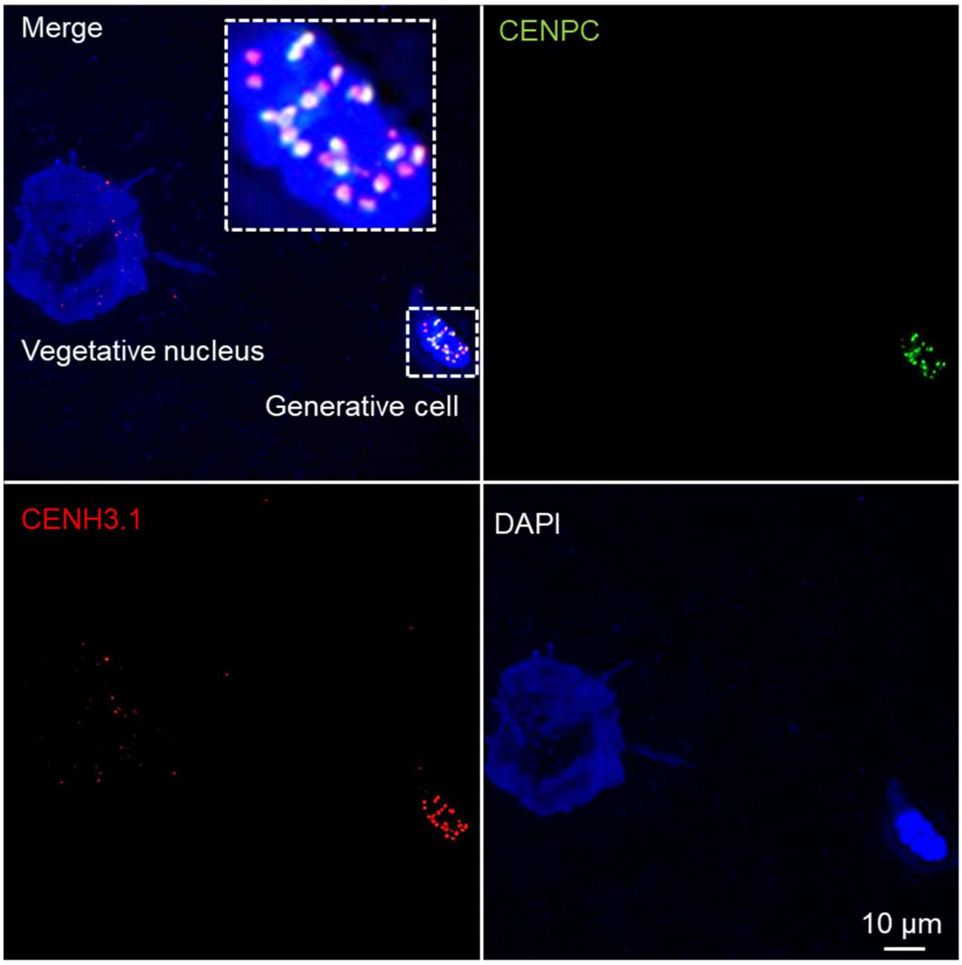

Supplementary Fig. 11: CENPC localization in matured pollen.

The generative nucleus of mature cowpea pollen shows colocalizing CENH3.1 (red) and CENPC (green)-specific immunosignals. The vegetative nucleus is free of centromeric immunomarks.

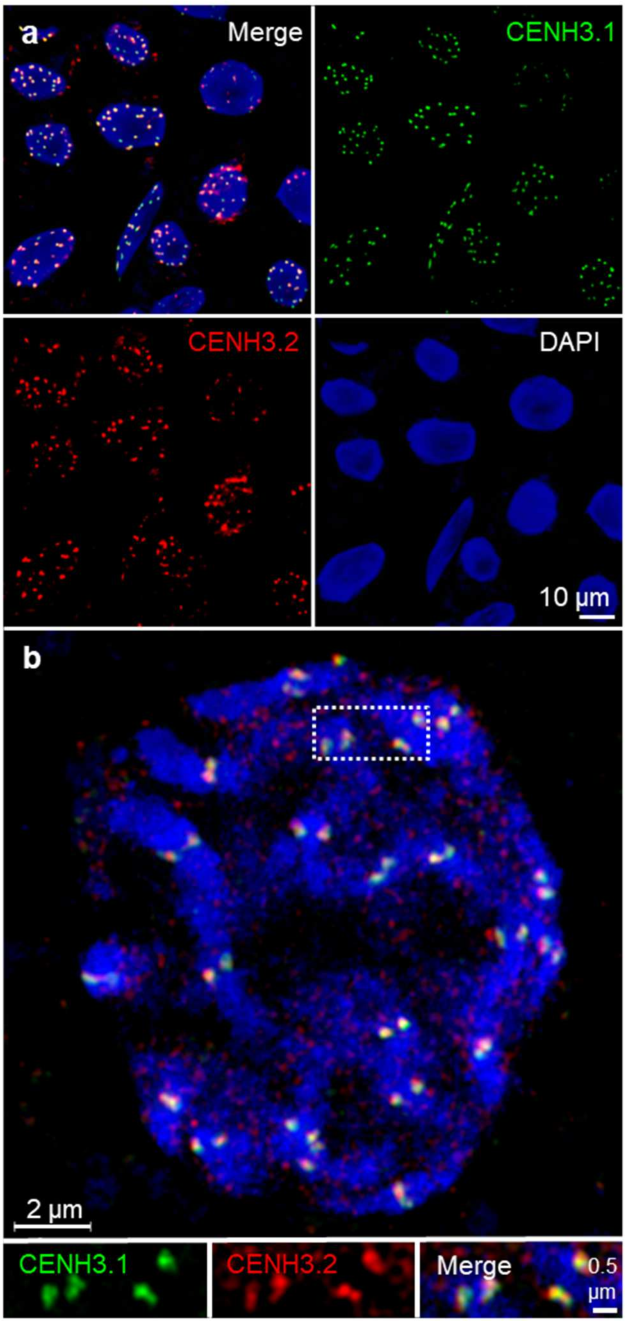

Supplementary Fig. 12: The centromeres of embryonic cells at early heart stage contain CENH3.1 and CENH3.2.

a and **b** CENH3.1 (green) and CENH3.2 (red) immunosignals colocalize in interphase (**a**) and prometaphase (**b**) cells. Structured Illumination Microscopy by SIM shows that both CENH3 variants occupy different centromeric subdomains (further enlarged inserts).
